# Viewing Landscapes Is More Stimulating Than Scrambled Images After a Stressor: A Cross-disciplinary Approach

**DOI:** 10.3389/fpsyg.2019.03092

**Published:** 2020-01-24

**Authors:** Mikaela Law, Gregory Minissale, Anthony Lambert, Urs M. Nater, Nadine Skoluda, Nathan Ryckman, Lenore Tahara-Eckl, Martina Bandzo, Elizabeth Broadbent

**Affiliations:** ^1^Department of Psychological Medicine, University of Auckland, Auckland, New Zealand; ^2^Department of Art History, University of Auckland, Auckland, New Zealand; ^3^School of Psychology, University of Auckland, Auckland, New Zealand; ^4^Faculty of Psychology, University of Vienna, Vienna, Austria

**Keywords:** nature, artwork, stress, fatigue, cortisol, pupil size

## Abstract

Research has demonstrated that nature is beneficial for many aspects of one’s health. This pilot study aimed to investigate whether viewing landscape artworks, as a form of representational nature, could improve psychological and physiological recovery from a laboratory stressor. A sample of 30 participants was randomized to one of two conditions: landscape and scrambled. After a laboratory stress task, participants in the landscape condition viewed a series of landscape paintings for 30 min; participants in the scrambled condition viewed digitally scrambled versions of these artworks as a control condition. Pupil size was measured while viewing the images using an eye tracker. Affect, drowsiness and fatigue, and the salivary stress biomarkers, cortisol, and alpha-amylase were measured at baseline, after the stressor, and after the artwork viewing period. After the viewing period, the scrambled condition had increased reports of low negative affect (which contains the variables of sleepy, dull, and sluggish) (*p* = 0.045, $\eta_{p}^{2}$ = 0.12) and increased reports of drowsiness (*p* = 0.038, $\eta_{p}^{2}$ = 0.12). Salivary cortisol levels decreased more rapidly while viewing the scrambled images compared to the landscape artworks (*p* = 0.027, $\eta_{p}^{2}$ = 0.62). Lastly, pupil size while viewing the landscape artworks was larger than when viewing a blank screen (*p* = 0.025, $\eta_{p}^{2}$ = 0.33), an effect not seen in the scrambled condition. This pilot study suggests that viewing landscape artworks was more stimulating and reduced drowsiness after stress when compared to viewing scrambled images.

## Introduction

Throughout history, exposure to nature has been touted as restorative for health and research supports this proposition. Although nature is experienced in multi-sensory ways, research has focused on the effects of viewing nature on health, due to the dominance of vision in experiencing nature (Ulrich, 1981).

Viewing nature has been found to have effects on health in both healthy and patient populations. One review found that the effects of nature fall into three categories: short-term recovery from stress, recovery from illness, and long-term improvements in mood (Velarde et al., 2007). Viewing nature, either live or through photographs and videos, has been linked with a wide range effects, including increased relaxation as shown through EEG (Chang, 2002), decreased anxiety about surgery (Ulrich et al., 1993), increased heart rate variability (Gladwell et al., 2012), decreased job stress, increased life satisfaction (Kaplan et al., 1988), and decreased pain (Vincent et al., 2010; White et al., 2019). A recent study by White et al. (2019) found that the optimal dose for improved health and well-being was 120 h of nature contact per week; however, the beneficial effects of nature have been found to occur within less than 5 minutes (Ulrich, 1992).

Research has also shown that nature has a restorative effect on the stress response. Stressed individuals report improved mood after viewing nature scenes compared to viewing nothing or urban scenes (Ulrich, 1979). Nature can also improve physiological recovery from a stressor when viewed after (Ulrich et al., 1991) or before (Brown et al., 2013) experimental stressors. Lastly, nature has also been found to lower cortisol levels in both stressed and unstressed individuals (Olafsdottir et al., 2018; Hunter et al., 2019).

Three main theories have been proposed for the beneficial effects of viewing nature on health; evolutionary theory, attention restoration theory (ART), and nature as positive distraction (Nanda et al., 2010). Evolutionary theory proposes that responses to nature are influenced by genetics (Kweon et al., 2008). As humans evolved in natural environments, we have an innate predisposition to experience restoration as a response to nature (Ulrich et al., 2003). As a consequence of our evolutionary heritage, natural environments are processed more efficiently, as our sensory, cognitive, and emotional systems evolved in this landscape (Ulrich et al., 1991). Conversely, these systems are likely to function more poorly in artificially constructed, urban environments that are more recent phenomena. This theory explains why natural scenes are more beneficial to health than urban scenes.

ART postulates that stress causes mental fatigue which impacts cognitive processes. However, the restorative characteristics of nature can counteract this, resulting in better recovery from this mental fatigue (Kaplan, 1995). In this theory, nature is restorative because it is attention-grabbing and engaging, in a non-threatening way, and therefore, reduces cognitive strain (Berman et al., 2008). ART therefore provides a possible explanation as to why people exposed to nature have reduced stress responses.

The last theory is that nature is a form of positive distraction. Positive distraction refers to an element of the environment that produces positive feeling and holds attention effortlessly. This attracts attention away from negative stimuli and experiences such as stress. Nature is effective as a positive distraction because it is stimulating and evokes interest and positive affect, allowing the displacement of negative affect (Hartig et al., 2011).

These three theories are not mutually exclusive. The attention system of the human brain (Posner and Petersen, 1990; Petersen and Posner, 2012) has a long evolutionary history (Graziano, 2014) and includes a range of processes, which include focusing attention, managing cognitive resources, and responding to distractions. Research to date shows strong evidence that viewing nature is beneficial to health and aids in stress recovery. There is also growing evidence indicating that representations of nature through artwork can have similar effects through the same mechanisms (Ulrich et al., 2006).

Artworks that depict nature scenes have increasingly been used in research and healthcare settings to reduce stress and improve health. Research demonstrates that, like real views of nature, artwork can reduce anxiety (Binnie, 2010), reduce depression (Staricoff et al., 2003), improve mood (Karnik et al., 2014), increase relaxation (Wang et al., 2015), and decrease anxiety medication usage (Nanda et al., 2011) when compared to no artwork. This collection of studies demonstrates that nature artworks can significantly improve psychological well-being. However, some research shows null results. A recent study found that nature artworks did not improve mood, pain, anxiety, depression, and satisfaction for chemotherapy patients (George et al., 2017). But in subsequent interviews, the patients reported the artwork provided a positive distraction from chemotherapy.

Although this evidence indicates that nature artworks have a positive effect on psychological health, there is a scarcity of evidence for the effects of these artworks on physiological outcomes. An early study by Heerwagen (1990) found that heart rate was lower for dental patients on the days where a landscape mural was hung in the waiting room compared to no mural. Mastandrea et al. (2019) found that visiting art museums lowered systolic blood pressure compared to visiting an office with no artworks; however, this study did not look specifically at artworks depicting nature. Therefore, more research is needed to determine whether experiencing nature artworks can improve physiological outcomes.

Most nature research has used urban scenes as a control. However, this approach has many confounding variables such as the degree of color in the nature vs. urban scenes, as color is an important mediator in the relationship between art and mood (Lankston et al., 2010). A more appropriate control is scrambled images, as used in previous research on the effects of artwork on attention and memory (Wang et al., 2015). Scrambled images are edited versions of artworks that have been digitally disarranged. We propose that these images act as better controls as they retain the colors and brightness of the original artwork, but the representation of nature is removed (Wang et al., 2015). This allows a better understanding of whether the depiction of nature in the art is the key factor in improving outcomes, rather than structural features.

The current pilot study used an interdisciplinary approach to investigate whether nature artworks can improve psychological and physiological recovery from a laboratory stressor, when compared to viewing scrambled images of the artworks. Stress responses were assessed using measures of: self-reported affect, fatigue and drowsiness, and salivary cortisol and alpha-amylase. Participants’ pupil size was also measured while viewing the artworks to provide an indication of the degree of stimulation and arousal that the artworks provided.

In this pilot study, we investigated the feasibility of study procedures, as well as estimates of effect size. Based on prior research, it was hypothesized that viewing nature artworks after a laboratory stressor would lead to improved stress recovery, as indexed by decreased salivary cortisol, alpha-amylase, fatigue and drowsiness, improved affect levels, and increased pupil size compared to the control condition who viewed scrambled images.

## Materials and Methods

### Sample

A sample of 30 adults (20 female, 10 male; average age 27.20 years, age range 18–52 years) was recruited from the community through flyers and email advertisements. Participants were included if they were over the age of 16 and spoke English. Ethics approval was granted by the University of Auckland Human Participants Ethics Committee.

### Procedure

In accordance with salivary sampling procedures, participants were instructed not to chew gum or drink caffeine, juice, or alcohol 18 h prior to the study and not to eat or brush their teeth in the hour before their session. Prior to their laboratory session, participants were randomized to one of two conditions: control (scrambled images) or nature (landscape images). Randomization was performed by a researcher uninvolved in the experiment using a random number generator. Randomization was concealed until the start of the session, when the sealed envelope containing the group allocation was opened.

**Figure 1:**
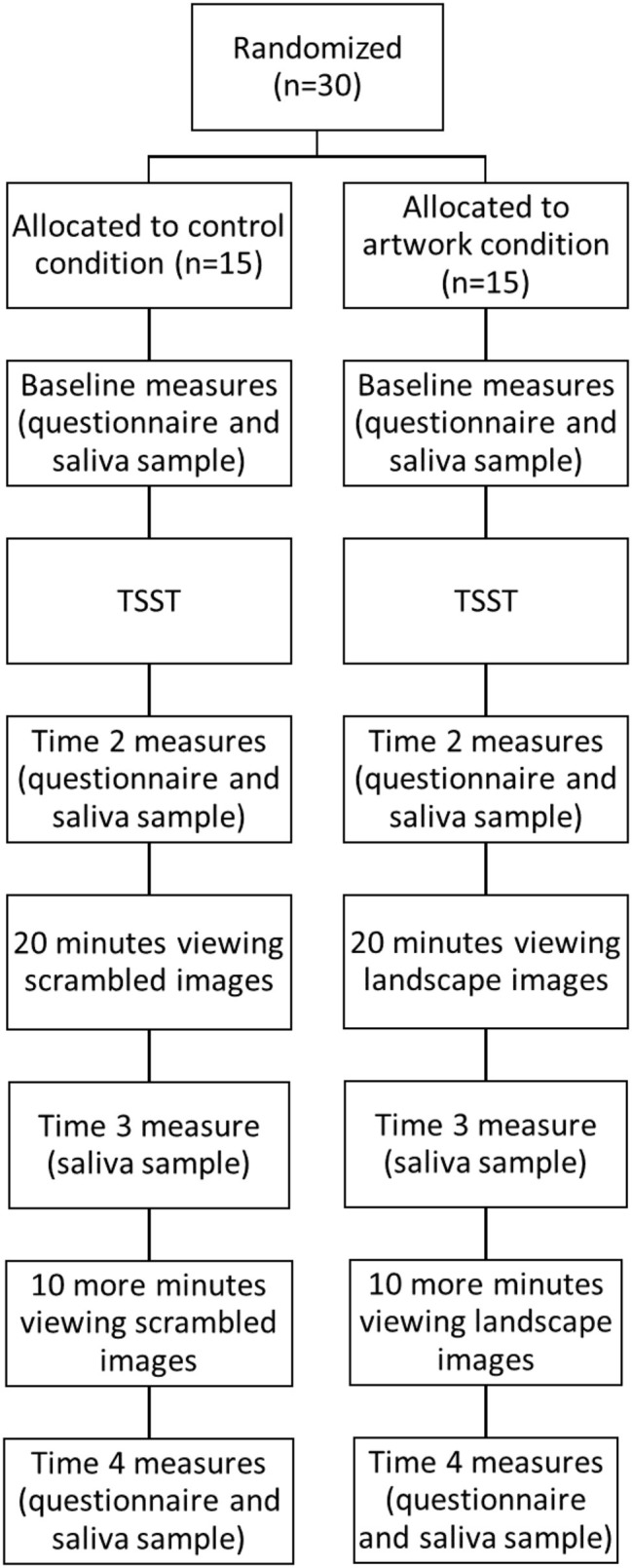
Flow chart showing the procedure of the study.

Participants attended a 90-min experimental session and gave written informed consent. The procedure is shown in Figure 1. Baseline questionnaires about the participant’s demographics and affect levels were completed, and the participant provided a saliva sample. Once baseline measures were taken, participants were exposed to a shortened version of the Trier Social Stress Test (TSST; Kirschbaum et al., 1993). Participants were given 3 min to prepare and 3 min to present a speech to convince the experimenter to give them their dream job. Participants were told their speech would be recorded and a panel of judges would review it and award the best speech with a $100 voucher. A shortened version of the TSST was used as a recent meta-analysis has shown that the shortened version produces similar physiological stress responses to the full TSST paradigm (Goodman et al., 2017).

**Figure 2:**
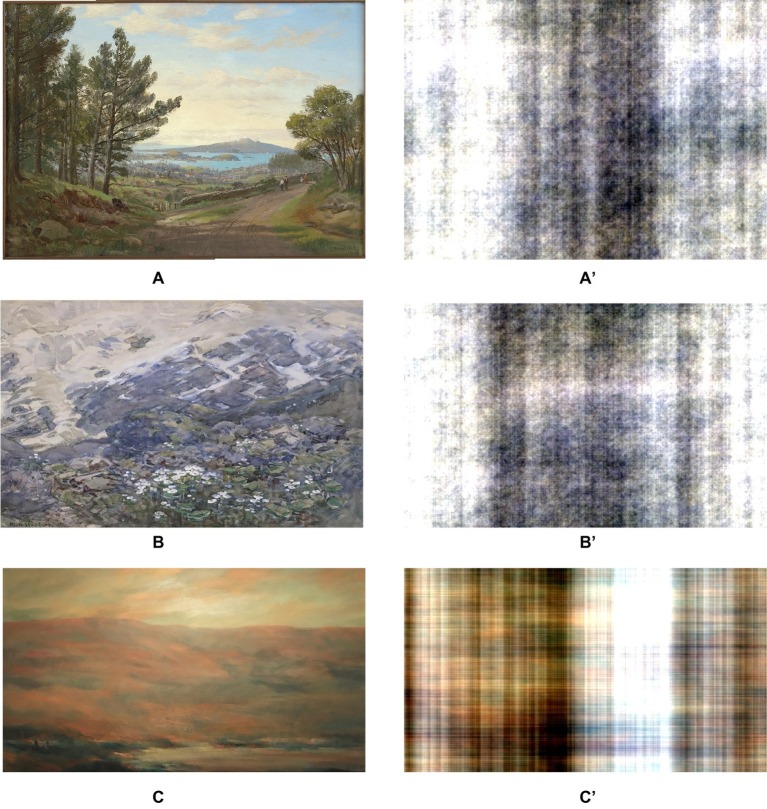
Examples of the landscape artworks **(A–C)** and their scrambled versions **(A′–C′)**. **(A)** is a painting by Charles Blomfield, Auckland Harbour from Mt Eden, which is in the public domain. **(B)** is by Margaret Stoddart, Mountain Lilies, reprinted with permission by the Christchurch Art Gallery Trust Collection. **(C)** is by Elizabeth Rees, Cove, reprinted with permission provided by Elizabeth Rees.

The participants then completed the affect measures and provided a saliva sample for a second time. They then viewed a 30-min slide-show comprising of 26 images based on their random group allocation. Participants in the landscape condition were shown a set of landscape artworks by New Zealand artists. The landscapes were included if they contained a nature scene that was relatively void of detailed focal points and non-natural stimuli. The scrambled condition viewed digitally scrambled versions of the landscape artworks, similar to work by Wang et al. (2015). A two-dimensional Fast Fourier transform was performed on each image, in order to generate the scrambled version. These images no longer have any sense of “objectness,” but they preserve the color and luminance profiles of the original images. Examples of the original artworks and their scrambled versions are shown in Figure 2. Participants’ pupil size was tracked during the viewing period.

Twenty minutes into this viewing period, the researcher entered and asked the participant to provide another saliva sample. Participants viewed the images for a further 10 min before completing the final set of measurements. At the end of the session, participants were debriefed and received a $40 voucher for participation.

### Measures

#### Demographics

At baseline, participants were asked about their demographics including: gender, age, height, weight, and ethnicity.

#### Affect

Affect levels were assessed at baseline, after the stressor, and after the viewing period using a modified version of the Actual Affect Subscale of the Affect Valuation index (AVI; Tsai et al., 2006). This scale consisted of a list of 25 emotions, and participants were asked to rate how much they felt that emotion at that present moment on a scale of 1 (not at all) to 5(extremely). These instructions were modified from the original scale, which asked the participants to rate how they felt over a typical week. These modified instructions have been used successfully in previous studies (Nair et al., 2015; Robinson et al., 2017).

Eight aggregate component scores were calculated: high arousal positive affect (HAP; strong, excited, and enthusiastic), low arousal positive affect (LAP; calm, relaxed, rested, and peaceful), positive affect (PA; happy, content, and satisfied), negative affect (NA; sad, lonely, and unhappy), high arousal negative affect (HAN; hostile, fearful, and nervous), low arousal negative affect (LAN; dull, sleepy, and sluggish), low arousal affect (LA: quiet, still, and passive), and high arousal affect (HA; aroused, surprised, and astonished). This scale is valid and reliable across different populations, and each component score has high internal consistency (Tsai et al., 2006).

#### Fatigue and Drowsiness

Participants were asked to rate how much they were feeling fatigue and drowsiness on a scale from 0 (not present) to 3 (severe) at baseline, after the stressor, and after the viewing period (Petrie et al., 2014).

#### Salivary Stress Biomarkers

Saliva samples were collected at baseline, after the stressor, 20 min into the viewing period, and after the viewing period as per protocol using SaliCaps collection device (IBL, Hamburg, Germany). These samples were taken to examine any changes in stress biomarkers (salivary cortisol and alpha-amylase) associated with the viewing and stress tasks (Strahler et al., 2017). Participants were asked to rinse their mouths with water, before collecting saliva using the passive drooling technique. Participants collected their naturally secreted saliva in their mouths for 2 min by not swallowing, before transferring the accumulated saliva to the SaliCap. The samples were stored at −20° C at the University of Auckland before they were shipped on dry ice to the University of Vienna, where they were biochemically analyzed. Concentrations of salivary cortisol were measured using commercially available enzyme-linked immunosorbent assay (ELISA, IBL, Hamburg, Germany). Salivary alpha-amylase activity was determined using a kinetic colorimetric test (Strahler et al., 2016) using reagents obtained by Roche (Roche Diagnostics, Mannheim, Germany). Intra- and inter-assay coefficients of variance of both tests were below 10%.

#### Pupil Size

Participants viewed digital versions of the artworks and scrambled images on a 23″ monitor, controlled by a Dell Optiplex PC. Viewing distance from the screen was approximately 60 cm, and changes in pupil size were monitored by an eye tracker (the EyeTribe, Denmark). Participants rested their chin on a chin rest, to keep their heads still during the viewing period. During the first (20 min) block of images, participants viewed 17 images in succession; in the second (10 min) block of images, participants viewed nine images. Each trial began with presentation of a dark screen with white cross in the centre for 3 s, followed by a uniform gray screen for 4 s, followed by an image (landscape or scrambled image) for 60 s. Participants were instructed to look at the white cross at the beginning of every trial. Following the disappearance of the cross, participants were free to move their eyes to explore the succeeding images. Each participant viewed a different random sequence of images, and allocation of images to trial blocks was counterbalanced.

### Statistical Analysis

Data were analyzed using IBM SPSS Statistics 22. Mixed factorial ANOVAs were completed to analyze the interaction and main effects of time-point (baseline, post-stressor, during artwork, and post-artwork viewing) and condition (scrambled vs. landscape) on affect, cortisol, and alpha-amylase. ANCOVAs for changes in cortisol and alpha-amylase controlling for baseline levels were conducted for the recovery period (from post-stressor to post-viewing period). The cortisol and alpha-amylase data violated the assumption of normality and was transformed using a natural log transformation and logged values were used in the analyses. Mean pupil size data (in mm^2^) were entered into a mixed factorial ANOVA with image (gray screen vs. image) and trial block (one vs. two) as within subjects factors; group (landscape vs. scrambled) was the between-subjects factor.

All tests were reported using the Greenhouse-Geisser adjustment due to violations in sphericity (Vasey and Thayer, 1987). All significant interaction effects were followed up using simple pairwise comparisons with Bonferroni corrections.

## Results

Baseline characteristics are given in Table 1. No significant differences between the two conditions were found.

**Table 1:** Summary of demographic and baseline characteristics of participants across condition.

Baseline variable	Scrambled	Landscape	*p*
Age (years) *M* (SD)	27.53 (8.83)	26.87 (5.41)	0.805^a^
Gender (%)			0.439^b^
Female	11 (73%)	9 (60%)	
Male	4 (27%)	6 (40%)	
Ethnicity (%)			1.000^b^
NZ European	6 (60%)	6 (60%)	
Non-European	9 (40%)	9 (40%)	
BMI *M* (SD)	24.50 (4.31)	25.28 (4.70)	0.640^a^
Exercise days/week, *M* (SD)	4.07 (1.75)	4.00 (2.20)	0.928^a^
Baseline salivary cortisol (nmol/L), *M* (SD)	3.61 (2.44)	4.01 (5.15)	0.778^a^
Baseline salivary alpha amylase (U/ml), *M* (SD)	81.92 (99.00)	44.49 (51.18)	0.204^a^

*M, mean; SD, standard deviation; %, percentage of participants in that category*.

*p was calculated by independent samples *t* tests^a^ and Chi-square tests^b^*.

### Affect

No significant main effects were observed for the effect of condition on any of the eight AVI component scores (all *p*’s *> 0*.05). Significant main effects of time-point were found for the following components: HAP [*F*_(2,53)_ = 21.33, *p* < 0.001, $\eta_{p}^{2}$ = 0.44], LAP [*F*_(2,46)_ = 6.38, *p* = 0.006, $\eta_{p}^{2}$ = 0.19], PA [*F*_(2,55)_ = 3.96, *p* = 0.025, $\eta_{p}^{2}$ = 0.12], HAN [*F*_(2,51)_ = 6.46, *p* = 0.004, $\eta_{p}^{2}$ = 0.19], LAN [*F*_(1,35)_ = 19.49, *p* < 0.001, $\eta_{p}^{2}$ = 0.41], LA [*F*_(2,53)_ = 7.04, *p* = 0.002, $\eta_{p}^{2}$ = 0.20], HA [*F*_(1,71)_ = 4.62, *p* = 0.029, $\eta_{p}^{2}$ = 0.14]. *Post hoc* tests indicated that irrespective of condition, the stressor caused PA and LAN to decrease, and the artwork viewing caused the low arousal affect components to increase and high arousal affect components to decrease. Therefore, irrespective of condition, the stress and viewing tasks change participants’ affect.

A significant interaction effect was observed on the LAN component [*F*_(1,35)_ = 3.97, *p* = 0.045, $\eta_{p}^{2}$ = 0.12]. Follow-up tests for each time-point revealed that there were no significant differences in LAN across the conditions at any time-point (all *p*’s *> 0.*05). However, the differences between conditions were approaching significance after the viewing period [*t_(28)_ =* 1.85, *p = 0*.075] with the scrambled condition having higher LAN affect (*M* = 8.33, SD *=* 3.42) than the landscape condition (*M* = 6.07, SD *=* 3.31).

A significant interaction effect was also observed on the HA component [*F*_(1,37)_ = 3.81, *p* = 0.048, $\eta_{p}^{2}$ = 0.12]. Follow-up tests revealed that there were no significant differences in HA across the conditions at any time-point (all *p*’s *> 0.*05). No significant interaction effects were observed for any other components.

ANCOVAs on change scores of the AVI components from post-stressor to post-viewing period, controlling for post-stressor scores, are shown in Table 2. LAN change scores across this period were significantly different between the two conditions with the control condition having a larger increase in LAN than the artwork condition.

**Table 2:** Differences between control and artwork conditions in mean change scores of the AVI component scores from post-stressor to post-viewing period, controlling for post-stressor scores (positive change scores indicate an increase in the parameter, while negative change scores indicate a decrease).

AVI component	Control, adj *M* (SD)	Artwork, adj *M* (SD)	*F*	df	*p*	$\eta_{p}^{2}$
High arousal positive	−2.19 (2.36)	−1.68 (1.39)	0.62	1, 27	0.440	0.02
Low arousal positive	2.13 (4.18)	2.41 (4.22)	0.04	1, 27	0.840	0.00
Positive	0.05 (2.41)	1.02 (1.44)	2.09	1, 27	0.160	0.07
High arousal negative	−1.16 (1.62)	−0.71 (1.18)	0.80	1, 27	0.380	0.03
Negative	−0.12 (0.83)	−0.08 (0.70)	0.02	1, 27	0.879	0.02
Low arousal negative	3.67 (3.11)	1.46 (2.42)	4.55	1, 27	0.042^*^	0.14
Low arousal	2.23 (2.26)	1.23 (2.23)	1.61	1, 27	0.215	0.06
High arousal	−1.19 (2.31)	−0.48 (1.30)	2.92	1, 27	0.099	0.10

*adj M, adjusted mean change score (post-viewing period − post-stressor), controlling for post-stressor scores; SD, standard deviation*.

* *p < 0.05*.

### Fatigue and Drowsiness

Kruskal-Wallis tests were conducted to examine the effect of condition on fatigue and drowsiness after the viewing period. There was a significant difference in drowsiness between the conditions [*H_(1)_* = 4.30, *p = 0*.038, $\eta_{p}^{2}$ = 0.12], with the scrambled condition indicating more drowsiness (mean rank = 18.40) than the landscape condition (mean rank = 12.60). The difference in fatigue between conditions after the viewing period approached significance [*H_(1)_* = 3.64, *p = 0*.057, $\eta_{p}^{2}$ = 0.09] with the scrambled condition reporting more fatigue (mean rank = 18.23) than the landscape condition (mean rank = 12.77).

### Salivary Stress Biomarkers

No significant main effects were observed for the effect of condition on cortisol [*F*_(1,26)_ = 1.11, *p* = 0.302, $\eta_{p}^{2}$ = 0.17] or alpha-amylase [*F*_(1,26)_ = 1.53, *p* = 0.227, $\eta_{p}^{2}$ = 0.22]. A significant main effect of time was found for cortisol [*F*_(3,68)_ = 7.87, *p* < 0.001, $\eta_{p}^{2}$ = 0.98]. Follow-up polynomial contrasts indicate a significant linear trend [*F*_(1,26)_ = 18.03, *p* < 0.001, $\eta_{p}^{2}$ = 0.98], with cortisol decreasing over the course of the experiment in both conditions: baseline (*M* = 1.03, SD = 0.84), post-speech (*M* = 0.76, SD = 0.94), during viewing (*M* = 0.76, SD = 1.04), and post-viewing (*M* = 0.59, SD = 0.82). There was no significant main effect of time for alpha-amylase [*F*_(2,58)_ = 0.89, *p* = 0.427, $\eta_{p}^{2}$ = 0.21].

There were no significant interaction effects of time-point and condition on salivary cortisol [*F*_(3, 68)_ = 1.72, *p* = 0.176, $\eta_{p}^{2}$ = 0.40] or alpha-amylase [*F*_(2, 58)_ = 1.65, *p* = 0.197, $\eta_{p}^{2}$ = 0.36]. However, there was a significant difference between conditions in the quadratic trends in cortisol over the session [*F_(1,26)_* = 4.49, *p* = 0.044, $\eta_{p}^{2}$ = 0.53]. Follow-up contrast analysis showed the quadratic trend was significantly greater for the landscape condition (*M* = 0.37, SD = 0.78) than the scrambled condition (*M* = −0.19, SD = 0.61), indicating that the landscape condition had a significant inverted U-shape relationship where cortisol levels decreased from baseline, but increased again after the viewing period. The scrambled condition did not have this U-shaped relationship.

ANCOVAs for change scores across the viewing period (after the stressor until after the viewing period) controlling for baseline levels were conducted for cortisol and alpha-amylase. Change scores for cortisol over the viewing period showed a significant difference between conditions when controlling for baseline cortisol [*F*_(1,26)_ = 5.49, *p* = 0.027, $\eta_{p}^{2}$ = 0.62]. The scrambled condition had a significantly greater mean decrease in cortisol over the viewing period (*M* = −0.36, SD = 0.43) than the landscape condition, which stayed fairly stable (*M* = 0.02, SD = 0.37). This demonstrates that during the viewing period, the scrambled condition had a larger decrease in cortisol levels. Change scores over the viewing period showed no significant differences in alpha-amylase between conditions when controlling for baseline values [*F*_(1,26)_ = 0.22, *p* = 0.216, $\eta_{p}^{2}$ = 0.23].

### Changes in Pupil Size

**Figure 3:**
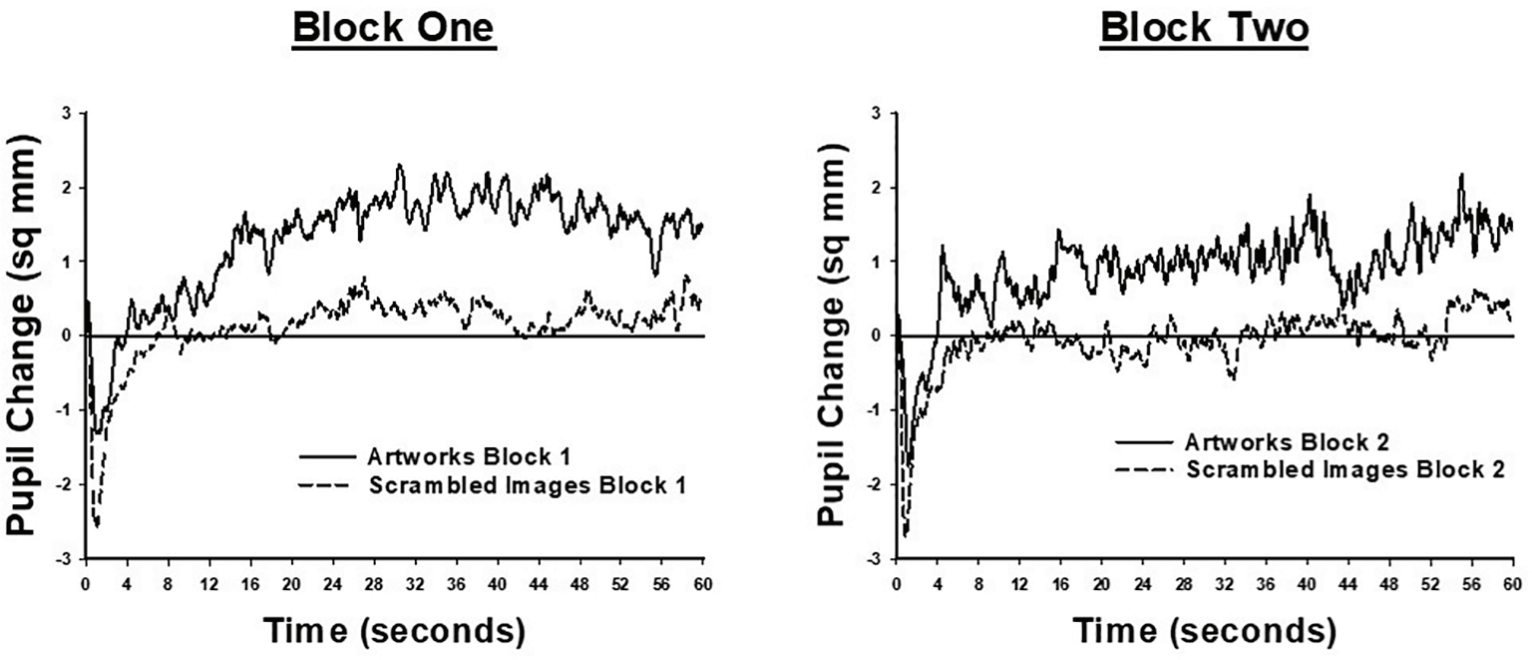
Moment to moment changes in pupil size, when participants viewed landscape artworks (solid lines) and scrambled images (dashed lines). Each plot shows the difference between pupil size at each time-point during the 60-s image viewing period and average pupil size when viewing the uniform gray screen presented before each image. The left-hand panel shows data from the first trial block, comprising 17 images; the right-hand panel shows data from the second trial block, comprising nine images.

Pupil size data were not available for one participant, in the landscape condition, due to technical difficulties. Results for the remaining participants are shown in Figure 3, which illustrates moment-to-moment changes in pupil size during the 60 s viewing period. Each plot shows the difference between pupil size at each viewing time-point, averaged across image trials, and mean pupil size, averaged across trials, during the 4 s of viewing a uniform gray screen, prior to each image. Data are shown separately for each block of viewing trials. As Figure 3 shows, in both blocks of trials, and for both conditions, image presentation was associated with a phasic pupil constriction within the first 1–2 s, followed by an extended period in which pupil size remained relatively constant. From very early in the trial (including during the phasic response to image onset) until the end of the viewing period, mean pupil area was larger for the landscape condition, relative to the scrambled condition. Following the initial phasic response to image onset, the pupils of the landscape condition remained larger than when viewing the uniform gray screen. In contrast, the pupil area of the scrambled condition remained similar to the mean pupil size recorded during the pre-image gray screen.

Statistical analyses confirmed this description of the pupil size findings. The main effect of trial block was significant [*F*_(1,27)_ = 35.43, *p* < 0.001, $\eta_{p}^{2}$ = 0.57] showing that mean pupil size was significantly larger in block two (*M* = 24.69, SE = 0.51) than block one (*M* = 23.10, SE = 0.48). The main effect of image was significant [*F*_(1,27)_ = 5.40, *p* = 0.028, $\eta_{p}^{2}$ = 0.17]; however, this effect was qualified by an interaction with condition [*F*_(1,27)_ = 4.74, *p* = 0.038, $\eta_{p}^{2}$ = 0.15]. This interaction was analyzed further by assessing effects of image separately for each condition. For participants in the landscape condition, pupil size was significantly larger when viewing a landscape artwork (*M* = 24.99, SE = 0.93) than when viewing a gray screen [*M* = 23.90, SE = 0.75, *F*_(1,13)_ = 6.41, *p* = 0.025, $\eta_{p}^{2}$ = 0.33]. For participants in the scrambled condition, pupil size when viewing a scrambled image (*M* = 23.36, SE = 0.56) and a gray screen (*M* = 23.32, SE = 0.51) did not significantly differ (*F* < 1, $\eta_{p}^{2}$ = 0.002). These results demonstrate that viewing landscapes led to an increase in pupil size which was not seen in the scrambled condition.

## Discussion

The aim of this research was to investigate whether viewing nature artwork in the form of landscape paintings could improve psychological and physiological responses after a stressor, compared to viewing scrambled versions of the artwork that blurred perceptual details. This pilot study was conducted to assess feasibility and estimated effect sizes and was not powered to detect significant effects. Nonetheless, there were some significant effects, and these results will inform a larger study.

Viewing the scrambled images compared to the landscape images led to an increase in low arousal negative affect (feeling dull, sleepy, and sluggish) and drowsiness. These subjective ratings are consistent with observations of pupil size. While viewing scrambled images, average pupil size was similar to when participants viewed a uniform gray screen, and smaller than when viewing landscapes. Physiological work has shown a remarkably close relationship between moment-to-moment changes in pupil size and activity in the locus coeruleus of the brain stem (Aston-Jones and Cohen, 2005; Joshi et al., 2016). The locus coeruleus plays a key role in the regulation of arousal. Accordingly, our observation of reduced pupil size is consistent with subjective reports that while participants viewed the scrambled images, they experienced feelings of drowsiness and low arousal.

This is the first study to examine the effects of scrambled images or artworks on cortisol. Contrary to our hypothesis, salivary cortisol levels decreased faster after viewing the scrambled images compared to the landscape artworks. Cortisol has been linked with higher alertness and lower fatigue (Tops et al., 2006), so these results suggest that people in the scrambled condition felt less stimulated. Again, these results are consistent with the pupil size findings, as increased pupil size has been associated with increased cognitive engagement, effort and increased arousal (Laeng et al., 2012; Sirois and Brisson, 2014). Taken together, these results suggest that the landscapes were more stimulating and engaged the viewers more than the scrambled images. This may be because artwork can be a form of visual environmental enrichment.

These preliminary results support theories and research on nature and demonstrate that these effects may translate to nature represented through art. The finding that landscape paintings led to less drowsiness, larger pupil size, and higher cortisol supports Kaplan’s (1995) ART theory which proposes that nature engages attention and therefore reduces the fatigue effects caused by stress. These results also agree with the research that has found that viewing nature leads to “wakeful relaxation” compared to viewing urban scenes (Ulrich, 1981).

These results also support Wang et al. (2015) who suggest that traditional Chinese paintings, where perceptual details are blurred, increased an “inward oriented” frame of mind inducing high levels of relaxation and mind wandering. This was in contrast to viewing realistic paintings that had the opposite effect and was occupied with a high level of attention and stimulation. Blurred images, which are plentiful in abstract art, traditional Chinese painting, and impressionism, could be examined in a larger study, with the aim of informing an effective and integrated multi-sensory approach to recovery. Therefore, the scrambled images may have acted similarly to abstract art and traditional Chinese paintings.

However, the findings that the landscape artworks increased stimulation also contradict previous research which demonstrated that viewing nature murals compared to no mural led to lower arousal as indicated by decreased heart rate (Heerwagen, 1990). However, this previous study was conducted with dental patients awaiting procedures, and therefore, the nature mural may have worked more as a distraction, rather than being restorative.

This study had a number of limitations. Most importantly, as a pilot study, this study had a small sample size which limited the power of the analyses to find significant effects. Future research should expand on this study with a larger and more diverse sample, including a larger diversity of ages and cultures. Secondly, the study was conducted in a laboratory setting which may have affected the ecological validity, making it difficult to generalize the results to everyday settings where artwork may be placed to improve health, such as in hospitals.

A further limitation was that the landscapes contained more realistic and recognizable features than the scrambled images. Therefore, the results may have been due to the realism of the artwork rather than the natural content. Future research should include urban landscapes and their corresponding scrambled images, as well as natural landscapes and their scrambled versions, to see whether realism or nature is the effective component.

Lastly, there was little indication that the TSST lead to a physiological stress response in participants with no increase in stress biomarkers observed. It may be that participants were not given enough time to acclimatize before taking the baseline saliva sample and were therefore feeling anxious at baseline. Also, the samples were taken around 15 min after the beginning of the stressful task. Research demonstrates that a peak in cortisol is expected at least 20 min after the onset of acute stress exposure, and therefore, this study may not have allowed enough time to sample the entirety of the physiological stress response (Dickerson and Kemeny, 2004; Kirschbaum and Hellhammer, 2007). Future research should allow for a longer sampling time after the stressor and the artwork viewing of at least 20–30 min to potentially ensure more reliable stress biomarker findings.

Research on the effects of viewing artworks on stress responses could consider multiple factors. These include content, perspective, color, composition, and level of abstraction. Research also needs to consider whether the art is viewed before or after a stressor. It would be difficult for one study to include all of these factors. This study compared landscape artworks with mostly natural content with a moderate level of realism to scrambled images after a stressor and found that the landscapes were more stimulating than the scrambled images. More research is needed to consider the role of other factors, such as those listed above.

## Conclusion

This pilot study gives an early indication that landscape artworks may reduce drowsiness and increase stimulation after stress compared to their scrambled images. We have yet to research whether the same results would be found for other types of artworks. This study sets up a framework to further explore these effects in a larger and more diverse sample. It is recommended that future research allow for a longer sampling time after the experimental tasks to be able to detect possible differences in salivary stress hormones, conduct the research in a more naturalistic setting, and use multiple control images. If certain kinds of artworks are found to be beneficial, this could inform their use in stressed populations.

## Data Availability Statement

The datasets generated for this study are available on request to the corresponding author.

## Ethics Statement

The studies involving human participants were reviewed and approved by the University of Auckland Human Participants Ethics Committee. The patients/participants provided their written informed consent to participate in this study.

## Author Contributions

ML, GM, AL, and EB contributed to the conception and design of the study. ML, LT-E, and MB ran the study procedures. ML statistically analyzed the psychological data. AL and NR statistically analyzed the pupil size data. UN and NS analyzed the saliva samples and statistical analysis of this data was done by ML. ML wrote the first draft of the manuscript. AL wrote sections of the manuscript. GM, AL, UN, NS, LT-E, and EB provided revisions to the manuscript. All authors provided approval for publication of this content.

### Conflict of Interest

The authors declare that the research was conducted in the absence of any commercial or financial relationships that could be construed as a potential conflict of interest.
